# Transfusion-associated capillary leak in cardiac surgery is linked to adverse postoperative outcomes: a prospective observational study

**DOI:** 10.1016/j.aicoj.2026.100040

**Published:** 2026-03-02

**Authors:** Babak Saravi, Ulrich Goebel, Jan O. Friess, Leonard Simeth, Melina Heine, Paul Philipp Heinisch, Zhide Meng, Lukas Wessendorf, Andrea U. Steinbicker, Felix Ulbrich, Jochen D. Muehlschlegel, Julian Hubrich, Jakob Wollborn

**Affiliations:** a Department of Anesthesiology, Brigham and Women’s Hospital, Mass General Brigham, Harvard Medical School, Boston, United States of America; b Department of Oral, Maxillofacial and Facial Plastic Surgery, Medical Faculty and University Hospital Düsseldorf, Heinrich-Heine-University Düsseldorf, Düsseldorf, Germany; c Department of Anesthesiology and Critical Care, Medical Center – University of Freiburg, Faculty of Medicine, University of Freiburg, Germany; d Department of Anaesthesiology and Pain Medicine, Inselspital, Bern University Hospital, University of Bern, Bern, Switzerland; e University of Cologne, Department of Anesthesiology and Intensive Care Medicine, Cologne University Hospital, Cologne, Germany; f Department of Congenital and Pediatric Cardiac Surgery, Technical University Munich, German Heart Center Munich, Munich, Germany; g Department of Anesthesiology and Critical Care Medicine, Johns Hopkins University School of Medicine, Baltimore, United States of America

**Keywords:** Cardiac surgery, Capillary leak syndrome, Transfusion, Fluid balance, Endothelial permeability

## Abstract

**Background:**

Cardiac surgery patients are prone to complex inflammatory reactions associated with increased microvascular permeability, edema formation, and Capillary Leak Syndrome. Perioperative transfusions have been independently associated with higher risks of complications. This study aimed to investigate the association between transfusion, Capillary Leak Syndrome, and adverse postoperative outcomes in cardiac surgery patients.

**Methods:**

A prospective observational cohort of 405 adults undergoing cardiac surgery was enrolled at a tertiary academic center from May 2019 to October 2020. Body impedance electrical analysis and serial serum biomarker measurements were conducted throughout the patients’ peri- and postoperative course. Main outcome measures included inflammatory and endothelial biomarkers (IL-6, IL-8, angiopoietin-2, syndecan-1), body fluid composition assessed by bioelectrical impedance, and clinical outcomes, including acute kidney injury (AKI), ICU length of stay (LOS), and ICU mortality. To study the impact of transfusion on outcomes, statistical analyses including logistic regression, case-matching, and exploratory clustering were performed.

**Results:**

Transfusions of red blood cells, fresh frozen plasma, or platelets were associated with elevated postoperative IL-6 (p < 0.001) and IL-8 (p < 0.001) levels, along with complications in a dose-dependent manner. Higher mortality was observed among patients receiving ≥5 units of each individual component. Transfusions were associated with modest increases in extracellular water and elevated serum levels of angiopoietin-2 and syndecan-1, consistent with Capillary Leak Syndrome, although absolute ECW differences were small. Cluster generation of transfusion-associated capillary leak (TAC) revealed that TAC patients were more frequently prone to death during their ICU stay (p = 0.006), had higher odds of developing AKI (p < 0.001), and a longer ICU LOS (p < 0.001).

**Conclusions:**

Among 405 patients undergoing cardiac surgery, perioperative transfusion was associated with higher inflammatory markers, an increase in extracellular water, and higher risks of AKI and ICU mortality in dose-response analyses, consistent with a transfusion-associated capillary leak phenotype that co-occurs with adverse postoperative outcomes.

## Introduction

In Europe, cardiac surgery remains among the highest consumers of allogeneic blood products, with transfused patients exhibiting higher complication and mortality rates [[1], [2], [3], [4]]. While transfusion of blood products may achieve the intended effect of assuring oxygen delivery in patients with bleeding and anemia, blood products have previously been associated with severe adverse effects, including transfusion-associated cardiac overload (TACO) and transfusion-related acute lung injury (TRALI) [[5], [6], [7], [8]]. These transfusion-associated complications may reflect a broader pathophysiological mechanism in which transfusions under pro-inflammatory conditions contribute to fluid shifts and capillary leakage [5,9]. For instance, in TRALI, a two-hit model has previously been proposed, in which an initial hit, such as systemic inflammation triggered through major surgery, primes the patient, and a secondary hit by the transfusion product results in severe pulmonary edema and increased mortality rates [5]. A recent publication defined a transfusion risk score of 24 preoperative items that predicts the risk of blood transfusion in patients [10]. However, after blood transfusions mechanistically a pathophysiological cascade evolves. The capillary leak syndrome (CLS) is a clinical entity often triggered by systemic inflammation and characterized by increased fluid extravasation, leading to intravascular hypovolemia, extravascular edema, and hypoperfusion [11]. Pathophysiologically, it has been associated with microvascular injury, glycocalyx shedding, and increased vascular permeability, resulting in fluid loss to third spaces, and has previously been linked to cardiac surgery with subsequent adverse outcomes [11,12]. Transfusions have been shown to increase pulmonary vascular permeability [13]. However, the interplay of various transfusion modalities, inflammation, CLS, and complications, especially as a result of blood transfusions, has not been examined in clinical practice yet. We hypothesized that transfusions are associated with CLS in cardiac surgery patients, contributing to increased postoperative complications and higher mortality rates.

## Methods

### Study design and population

This is a secondary analysis of an observational, multidisciplinary, prospective study [14]. Its design complied with the STROBE guidelines for observational studies. At the Medical Center of the University of Freiburg, Germany, patients were recruited before their cardiovascular surgery and were followed in the ICU postoperatively from May 2019 to October 2020. IRB approval was obtained (Freiburg, EK-Nr. 405/18) by the Ethics Committee of the University of Freiburg, Engelbergerstrasse 21, 79106 Freiburg, Germany on January 14, 2019, and complies with the tenets of the Declaration of Helsinki. The study was registered in the German Clinical Trials Registry (DRKS No. 00017057), and written informed consent was obtained from all participants. Parts of the study have previously been published [14].

Eligibility of all adult patients scheduled for cardiovascular surgery with the anticipated use of cardiopulmonary bypass (CPB) was assessed. Patients were excluded when refused to participate, already participated in other clinical trials with pharmacological interventions, or had one of the following conditions: infection with HIV, viral hepatitis, idiopathic capillary leak syndrome (“Clarkson’s disease”), hereditary C1-esterase deficiency, recurrent angioedema, pre-existing chronic kidney failure requiring dialysis, and/ or pre-existing hepatic impairment with a Model for End-Stage Liver Disease (MELD) score≥20, pertinent to the hypothesis of the initial study. Of the 918 screened patients for inclusion, the final study size (n = 405) reflected all consecutive eligible patients undergoing cardiac surgery during the recruitment period. Patients not meeting eligibility criteria were excluded, and follow-up was completed for all included patients until ICU discharge or death. Deidentified data and analytic code are available from the corresponding author upon reasonable request and subject to institutional approvals.

### Study measurements

Preoperatively, measurements were obtained after the patient was placed under general anesthesia, and after completion of the procedure, followed by daily measurements during the ICU stay. An electrical analysis of body impedance was conducted using the Nutriguard-MS (Data Input GmbH, Poecking, Germany) as previously described [12]. A NutriPlus software program was used to analyze the data (Data Input GmbH, Poecking, Germany). Angiopoietin-2, VE-Cadherin, syndecan-1, and ICAM-1 levels were measured from the patients' serum using enzyme-linked immunosorbent assay (ELISA), while cytokine levels were obtained using a fluorescence-activated cell sorting (FACS) assay. Measurements and biomarker assessments were performed via identical protocols across all patients to reduce measurement bias. Transfusions were recorded intraoperatively and through postoperative day 1; units were categorized as 0, 1–4, or ≥5. This timeframe was chosen to ensure temporal alignment with biomarker measurements and to minimize the risk of reverse causation. Transfusion thresholds were applied separately for each blood component type; ≥5 units refers to the total units of each individual component, not the combined total across all types. These categories were pre-specified based on clinically meaningful thresholds. For exploratory dose-response analysis, finer gradations (0, 1–4, 5–8, ≥9 units) were examined to assess linearity of associations. AKI was defined by Kidney Disease: Improving Global Outcomes (KDIGO) criteria within the first 7 postoperative days. Patients with pre-existing chronic kidney disease requiring renal replacement therapy were excluded per protocol. Renal replacement therapy initiation was recorded as a binary outcome without prospective categorization of the primary indication. ICU mortality was defined as death prior to ICU discharge. Bioimpedance analysis yielded the extracellular water (ECW)/total body water (TBW) ratio and absolute ECW; changes were calculated as Postoperative Day 1 (POD 1) minus preoperative values.

### Statistical analyses

Analyses were performed in Python and SPSS (v27). Continuous variables are reported as mean ± SD or median [IQR]; categorical variables as n (%). Normality was assessed with the Shapiro–Wilk test and evaluation of skewness. Variables with substantial skewness (>1) were reported as median [IQR]; variables with skewness <1 were considered approximately symmetric and reported as mean ± SD. Group comparisons used t-tests or Mann–Whitney U tests for continuous data and χ^2^ or Fisher’s exact tests for categorical data, as appropriate. For comparisons across >2 independent groups, one-way ANOVA or Kruskal–Wallis tests were applied. Cytokines and endothelial markers were analyzed as Δ(POD1–pre-op). To capture the global inflammatory response, individual cytokine values were standardized, their ranks averaged into a composite score, and patients were dichotomized into ‘high’ vs ‘low’ responders at the prespecified 75th percentile. This approach mirrors methods in prior interleukin studies where the 75th percentile cut-off has been used to identify a ‘high inflammation’ subgroup that shows stronger associations with clinical outcomes [15,16]. Moreover, dichotomizing at the upper quartile helps isolate patients with the most pronounced biomarker elevations, enhancing signal specificity in heterogeneous populations. Multiplicity across biomarkers was controlled with the Benjamini–Hochberg false discovery rate (q = 0.05). To explore determinants of high inflammatory response and assess whether transfusion associations were independent of illness severity and endothelial changes, we fitted a multivariable logistic regression with inflammatory panel alteration (high vs. low) as the dependent variable. Variables for multivariable logistic regression were selected a priori based on clinical relevance and biological plausibility. Covariates included baseline severity markers (EuroSCORE-II, left ventricular ejection fraction [LVEF] <30%, surgical category), transfusion exposure (Packed Red Blood Cells [PRBC], Fresh Frozen Plasma [FFP], and platelets entered separately), and mechanistic variables (changes in Ang-2, VE-cadherin, syndecan-1, ICAM-1, and ECW). PRBC transfusion was entered as a binary variable (any vs. none) in the model. Although surgical category and LVEF contribute to EuroSCORE-II, they were included as separate covariates to account for potential residual confounding, as the EuroSCORE-II was not designed to capture inflammatory or fluid balance-related outcomes. Collinearity among covariates was assessed using variance inflation factors (VIF); all values were below 2.5, indicating no problematic multicollinearity. To mitigate confounding by postoperative severity, we performed an exploratory Sequential Organ Failure Assessment (SOFA)-matched comparison (post-exposure) of deaths vs survivors. Finally, we applied two-step clustering to identify a capillary leak phenotype using two variables: (1) inflammatory panel status (dichotomized at the 75th percentile) and (2) absolute change in extracellular water (ΔECW, POD1 minus preoperative). Notably, transfusion exposure was not included in the clustering algorithm. Cluster number and stability were evaluated by the silhouette coefficient. The association between identified clusters and transfusion exposure was assessed separately using chi-square tests. The designation 'transfusion-associated capillary leak' (TAC) reflects the observed co-occurrence of transfusion with the inflammatory/fluid shift phenotype identified through clustering, not a presumption of causality. ECW data were available for 394 patients. Missing ECW data (<3%) resulted from technical artifacts in bioimpedance measurements. Given the low proportion and random mechanism, no imputation was performed. All other clinical and laboratory variables were complete without missing values (n = 405). P-values ≤ 0.05 were considered statistically significant.

## Results

### Descriptive statistics

Among the 405 patients included in this analysis (mean age 63 ± 14 years; 75% male), most patients underwent Coronary artery bypass grafting (25%), followed by aortic surgery (22%), and mitral valve surgery (13%), with a median CPB time of 135 (IQR: 105−165) min. Further details on the patient population are illustrated in Table 1, and the patient flowchart is shown in Supplemental File 1.

**Table 1:** Descriptive statistics of patient demographics.

		Mean ± std/Median (IQR)		Count (%)
Age		63 ± 14		
Sex	F			100 (24.7)
	M			305 (75.3)
Body mass index		27.0	4.9	
Surgery	Aortic valve			63 (15.6)
	Coronary artery bypass grafting			103 (25.4)
	Mitral Valve			52 (12.8)
	Aorta			89 (22.0)
	Left Ventricular Assist Device			21 (5.2)
	Others			77 (19.0)
Length of stay ICU		3 (1−5)		
Postoperative day of extubation		0 (0−1)		
Cardiopulmonary bypass time		135 (105−165)		
EuroSCORE-II		6 (3−9)		
SOFA-score		9 (8−11)		
Transfusion Packed Red Blood Cells	Any PRBC			157 (38.8)
	1−4			118 (29.1)
	5−8			24 (5.9)
	≥9			15 (3.7)
Transfusion Fresh Frozen Plasma	Any FFP			109 (26.9)
	1−4			75 (18.5)
	5−8			15 (3.7)
	≥9			19 (4.7)
Transfusion Platelets	Any platelets			164 (40.5)
	1−4			146 (36.0%)
	5−8			14 (3.5)
	≥9			4 (1.0)
Died	No			375 (92.6)
	Yes			30 (7.4)
Acute Kidney Injury	No			242 (59.8)
	Yes			163 (40.2)

### Association between transfusion, inflammation, and capillary leak

Patients receiving PRBC, FFP, or Platelet transfusions had markedly elevated postoperative cytokine levels compared to the preoperative course, with particularly pronounced increases in IL-6 (p < 0.001) and IL-8 (p < 0.001). There was only a significant increase for IL-1 beta in the PRBC group (p = 0.003) and no increase in TNF-alpha. To obtain more insight into the development of CLS, we analyzed circulating serum biomarkers previously associated with CLS. All transfusion groups exhibited a significant increase in Angiopoietin-2 (p < 0.001) and syndecan-1 levels, consistent with CLS [14]. A significant decrease in VE-cadherin levels was observed only in the PRBC transfusion cohort (Table 2).

**Table 2:** Association between transfusion and endothelial biomarker levels alterations, as well as the Association between transfusion and body water distributions are illustrated. P-values are shown for the comparisons of columns within transfusions.

	Transfusion PRBC					Transfusion FFP				Transfusion Platelets		
	No		Yes			No		Yes		No	Yes	
	Mean (95%CI)		Mean (95%CI)			Mean (95%CI)		Mean (95%CI)		Mean (95%CI)	Mean (95%CI)	
Angiopoietin2	3.47 (3.20−3.74)		5.50 (4.53−6.47)			3.66 (3.33−3.98)		5.88 (4.64−7.11)		3.41 (3.11−3.70)	5.50 (4.59−6.40)	
p-value	**<0.001**					**<0.001**				**<0.001**		
VE-Cadherin	−0.34 (−0.38-(−0.30))		−0.51 (−0.60-(−0.42))			−0.39 (−0.44-(−0.34))		−0.44 (−0.53-(−0.36))		−0.38 (−0.44-(−0.32))	−0.44 (−0.50-(−0.38))	
p-value	**0.001**					**0.437**				**0.079**		
Syndecan1	71.20 (58.23−84.17)		150.61 (94.65−206.56)			77.85 (63.67−92.03)		167.15 (90.10−244.20)		67.44 (54.03−80.86)	152.28 (99.35−205.20)	
p-value	**0.003**					**0.006**				**0.001**		
sICAM1	48.72 (24.16−73.27)		51.05 (32.67−69.42)			46.81 (26.00−67.61)		57.42 (32.11−82.73)		47.72 (21.92−73.51)	52.45 (36.72−68.17)	
p-value	**0.621**					**0.621**				**0.362**		
ECW ratio		1.39 (1.36−1.41)		1.47 (1.43−1.50)	1.41 (1.38−1.43)		1.45 (1.41−1.49)		1.39 (1.36−1.41)			1.46 (1.43−1.49)
p-value		**<0.001**			**0.037**				**<0.001**			
ICW/ECW ratio change		7.72 (4.15−11.28)		6.56 (2.57−10.55)	8.01 (4.66−11.35)		5.19 (1.27−9.13)		8.29 (4.69−11.91)			5.77 (1.81−9.73)
p-value		**0.003**			**0.017**				**0.171**			

To assess the association of the inflammatory and endothelial profile with fluid shifts, we analyzed intra- and extracellular water. All transfusion types were associated with a significant increase in ECW over time. Additionally, PRBC and FFP transfusions induced a shift from intracellular compartment (ICW) to the extracellular space. However, this association did not reach statistical significance for platelet transfusions (Table 2).

For a comprehensive insight into the pro-inflammatory profile of our patients, we constructed a categorical variable based on the pro-inflammatory in two groups (below and above the 75th percentile). All types of transfusions were significantly associated with inflammatory panel alterations over time, and high inflammation panel alterations over time were associated with an increase in ECW (p = 0.015) (Table 3). The increase in ICW/ECW change was not different (p = 0.111). Patients with a marked increase in the inflammatory panel alterations were more prone to death (p = 0.005). Notably, ECW increased more profoundly when patients had ≥5 units of each individual component transfused and concurrently were among the high inflammation group.

**Table 3:** Association between transfusion and inflammatory panel change.

			Inflammatory panel change			
		Low pro-inflammatory cytokine profile		High pro-inflammatory cytokine profile		
		Count (%)	Mean (95% CI)	Count (%)	Mean (95% CI)	p-value
Transfusion Packed Red Blood Cells	No	228 (67.7%)		19 (30.6%)		**<0.001**
	Yes	109 (32.3%)		43 (69.4%)		
Transfusion Fresh Frozen Plasma	No	258 (76.6%)		36 (58.1%)		**0.002**
	Yes	79 (23.4%)		26 (41.9%)		
Transfusion Platelets	No	214 (63.5%)		26 (41.9%)		**0.001**
	Yes	123 (36.5%)		36 (58.1%)		
ECW change			1.40 (1.38−1.42)		1.49 (1.43−1.56)	**0.015**
ICW/ECW change			6.14 (3.34−8.93)		13.54 (5.38−21.71)	**0.111**
Died	No	320(95.0%)		53(85.5%)		**0.005**
	Yes	17(5.0%)		9(14.5%)		

To assess whether transfusion was independently associated with inflammatory response after accounting for illness severity and endothelial changes, we performed multivariable logistic regression with inflammatory panel alteration as the dependent variable. The model included surgical category, EuroSCORE-II, LVEF < 30%, transfusion status (PRBC, FFP, and platelets), changes in CLS biomarkers (Ang-2, VE-cadherin, syndecan-1, ICAM-1), and ECW change. Variance inflation factors confirmed no problematic multicollinearity among covariates (all VIF < 2.5). PRBC transfusion remained independently associated with high inflammatory panel alteration (OR 2.63, 95% CI 1.18–5.87, p = 0.018), even after adjustment for illness severity and biomarker changes. EuroSCORE-II was also independently associated (OR 1.09, 95% CI 1.00−1.19, p = 0.043). Among CLS biomarkers, syndecan-1 change was independently associated with inflammatory response (OR 1.002, 95% CI 1.00−1.004, p = 0.012), while VE-cadherin change showed an inverse association (OR 0.35, 95% CI 0.15−0.84, p = 0.018). Ang-2, ICAM-1, and ECW changes were not independently associated. FFP transfusion (OR 0.80, p = 0.63), platelet transfusion (OR 0.83, p = 0.67), and surgical category (p = 0.32) showed no significant associations. Notably, among the three blood component types, only PRBC transfusion was independently associated with high inflammatory panel alteration, whereas FFP and platelet transfusion showed no significant associations, suggesting a component-specific effect. To show the interconnection between transfusion, inflammation, and CLS, we illustrate the effects in Supplemental File 2. The findings indicate that ECW increased more profoundly when patients had ≥5 units of each respective component type (PRBC, FFP, or platelets) and were among the high inflammation group with an increase of the inflammation panel above the 75th percentile.

### Transfusion and complications

**Fig. 1:**
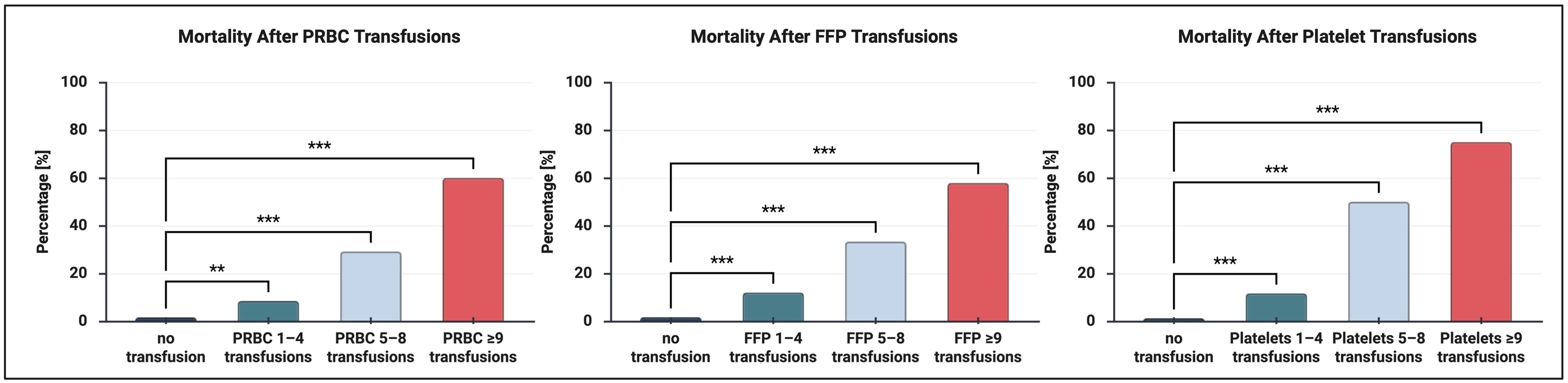
Illustration of the impact of transfusion amount on mortality. Significance symbols indicate pairwise comparisons versus 'no transfusion' group. *p < 0.05, **p < 0.01, ***p < 0.001, ns = not significant. Created in BioRender. Hubrich, J. (2026) https://BioRender.com/pao3du1.

Evaluating the association between the amount of PRBC, FFP, and Platelets given to the patients and the mortality observed, a significantly higher mortality rate was observed for an increasing amount of PRBC and FFP. Similar associations were observed for AKI and Length of Stay (LOS) (Figs. 1 and Supplemental File 3). The mortality rates started to differ significantly from the non-transfusion group when ≥5 units of each individual component (e.g., ≥5 units of PRBC, ≥5 units of FFP, or ≥5 units of platelets) were given.

Our results show significant differences in LOS between the transfusion amounts for all three transfusion types (p < 0.001). In exploratory dose-response analyses using finer gradations (0, 1–4, 5–8, ≥9 units), post-hoc pairwise comparisons for all transfusion types showed that receiving “no transfusion” was significantly different from 1−4, 5−8, and ≥9 transfusion amounts (p < 0.0001), with a shorter LOS in the no transfusion group. Furthermore, 5−8 transfusions revealed significantly higher inflammation than 1−4 transfusions for the FFP (p = 0.002) and PRBC (p = 0.028) transfusions.

To evaluate whether the association of transfusion and mortality was due to patients receiving transfusions having higher morbidity postoperatively, we applied a case-matching technique utilizing the postoperative SOFA score to match patients. The matching resulted in n = 29 patients who died during ICU and n = 29 patients with identical SOFA scores who did not die during ICU stay. Despite matching for the SOFA score, patients who received transfusions were significantly more prone to death during their ICU stay.

### Transfusion-associated capillary leak and complications

To explore the combined impact of transfusion, inflammation, and fluid shifts, we applied two-step clustering using transfusion exposure, inflammatory panel status, and perioperative ECW changes. The algorithm identified two distinct clusters (silhouette coefficient 0.8). Interestingly, Cluster 1 comprised exclusively patients with a high inflammatory response (n = 62), while Cluster 2 included exclusively patients with low inflammatory response (n = 332). An additional 11 patients could not be assigned to either cluster and were classified as outliers; they were not included in the main comparison. These patients had a mean age of 66.36 ± 8.86 years, 64% were male, 27% developed AKI, and 36% died during ICU stay. Cluster 1 was characterized by a higher mean ECW increase (9.3 ± 4.3 vs. 7.8 ± 4.0), a greater transfusion burden across PRBC, FFP, and platelets (all p < 0.001), and was therefore designated as the transfusion-associated capillary leak (TAC) group.

**Fig. 2:**
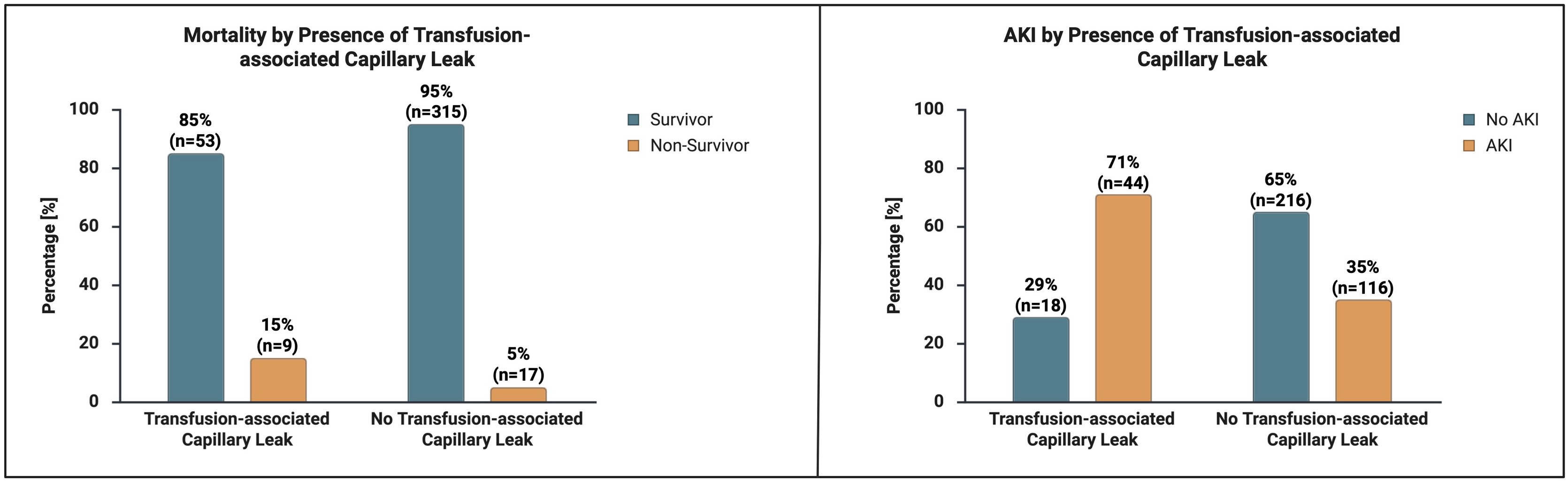
Comparison between patients with transfusion-associated capillary leak (TAC) and non-TAC patients with regard to mortality and acute kidney injury (AKI). TAC was identified by unsupervised clustering based on inflammatory response and extracellular water changes; the designation 'transfusion-associated' reflects co-occurrence with transfusion exposure and does not imply causation. Mortality and AKI were significantly more frequent in TAC patients compared to non-TAC patients (mortality p = 0.006; AKI p < 0.001). Created in BioRender. Hubrich, J. (2026) https://BioRender.com/gtwyfcf.

Clinical outcomes differed significantly between groups. Mortality during ICU stay was higher in the TAC group (14.5% vs. 5.1%, p = 0.006). AKI occurred more frequently in TAC patients (71.0% vs. 34.9%, p < 0.001). Moreover, ICU length of stay was markedly prolonged in TAC patients (median 5 [IQR 3–10] vs. 2 [IQR 1–4] days, p < 0.001). These findings suggest that TAC, defined by transfusion exposure combined with marked inflammatory activation and ECW expansion, identifies a subgroup of cardiac surgery patients at substantially higher risk of adverse outcomes (Fig. 2).

## Discussion

The presented findings suggest that all types of transfusions (PRBCs, FFPs, and platelets) were associated with higher rates of complications and stronger inflammatory responses. In patients undergoing cardiac surgery, transfusions post-cardiac surgery were significantly associated with CLS and severe postoperative complications. This study aimed to delineate the association between blood transfusion, hallmark features of CLS, and subsequent adverse outcomes.

Inflammatory disruption of the endothelial barrier and the resulting increase in vascular permeability have been identified as central components in the development of CLS [11]. Endothelial injury can result in a loss of structural integrity and promote the transition of endothelial cells into an activated, proinflammatory state, characterized by the release of cytokines such as interleukins [11,17,18]. While this response may support immune defense through enhanced leukocyte trafficking via vascular leakage on a local level, an escalation to a systemic level can lead to a widespread compromise of the endothelial barrier, resulting in CLS and associated complications [11,19,20]. IL-6 and IL-8 have previously been identified as markers of CLS [12]. In our cohort, transfusion was associated with a pronounced increase in IL-6 (p < 0.001) and IL-8 (p < 0.001), suggesting a mechanistic contribution to CLS development and supporting their role as diagnostic indicators.

Transfusions were associated with statistically significant but modest increases in ECW ratio (Δ0.04–0.08) and decreased ICW/ECW ratio. While these findings are consistent with fluid extravasation, the small absolute differences and substantial overlap between groups limit conclusions about clinical significance. However, this finding contradicts the common belief that blood products with their oncotic properties result in a longer-lasting effect on circulating intravascular volume compared to crystalloid fluid administration. The rate of fluid extravasation is altered by the inflammatory milieu of patients. Our findings demonstrate that transfusion is associated with an enhanced pro-inflammatory response, which in our patients is associated with further fluid extravasation and clinical deterioration. Transfusions also may exacerbate inflammation, contributing to CLS through further compromise of the endothelial barrier and increased vascular permeability [11,[21], [22], [23]]. Oya et al. revealed that patients who received intraoperative PRBC transfusions experienced higher ECW values, which is in agreement with our results, although patients underwent esophagectomy [24]. In addition, we observed a decrease in the ICW/ECW ratio among transfused patients in comparison to those who were not transfused. Capillary leak and mortality in critically ill patients are associated with decreasing ICW/ECW ratios [25].

This study further expands the knowledge on the consequences of profound inflammation and CLS by examining not only fluid shifts and systemic inflammation but also elucidating other indicative biomarkers of CLS, including glycocalyx shedding and upregulation of pro-permeability markers [12]. In our study, transfusions were associated with significantly elevated levels of Angiopoietin-2 and syndecan-1, hallmarks of CLS. Angiopoietin-2 was previously shown to play a key role in cytokine-induced vascular leakage and has been associated with AKI and increased LOS in the ICU after cardiac surgery [14,26]. This adds to our previous analysis of angiopoietin-2 in the same cohort by placing endothelial activation into the broader context of perioperative transfusion and systemic inflammation, supporting a multifactorial concept of CLS, in which transfusion exposure and elevated Angiopoietin-2 levels may represent associated markers of CLS [14]. CLS has also been linked to glycocalyx shedding, particularly to syndecan-1, which plays a relevant role in controlling vascular permeability [[27], [28], [29]]. Previous studies suggested that elevated plasma concentrations may indicate glycocalyx shedding and endothelial damage [29]. Consistent with this, transfusions were associated with features resembling the pathophysiological hallmarks of CLS, including systemic inflammation, fluid shifts to third spaces, and elevations in Angiopoietin-2 and syndecan-1.

Patients with TAC were identified via inflammatory panel and ECW alterations in this study, with these patients exhibiting increased mortality rates (p = 0.006), more frequent AKI (p < 0.001), and longer LOS (p < 0.001). For TRALI, one of the leading causes of transfusion-related deaths, a two-hit model has been proposed [5]. Here, a first hit, caused by surgery with a subsequent systemic inflammation, may prime the patient for a second hit, such as transfusions, resulting in increased permeability edema, acute respiratory distress, and higher mortality rates [5]. In cardiac surgery, the systemic inflammatory response triggered by the surgical trauma and CPB may lead to a breakdown of the endothelial barrier and thereby an increase in capillary permeability, resulting in fluid shifts into the extracellular compartment and CLS [11]. In analogy to TRALI, our cohort showed elevated IL-6 and IL-8 levels, reflecting systemic inflammation during the initial first hit phase [5]. Transfusions may then act as a second hit, amplifying the underlying pathophysiological cascade by further promoting systemic inflammation, endothelial damage, and increasing microvascular permeability. In TRALI, trapped polymorphonuclear neutrophils within the lung capillary bed are activated by anti-leukocyte antibodies and biological response modifiers from transfused blood components, leading to the production and release of reactive oxygen species [[30], [31], [32]]. This damages the endothelial barrier, resulting in capillary leakage and pulmonary edema [[30], [31], [32]]. The pathomechanism also occurs in CLS, with an inflammatory breakdown of the endothelial barrier and glycocalyx shedding further aggravating an increase in systemic inflammation, endothelial damage, and enhanced microvascular permeability [11]. This may further exacerbate CLS and contribute to increased mortality rates and longer ICU LOS. Notably, patients with TAC were also prone to AKI. When fluid infusion exceeds the lymphatic drainage capacity in the microcirculation, interstitial edema can occur in all organs including kidneys, resulting in reduced renal arterial blood flow, venous return, and lymphatic drainage, resulting in tissue hypoxia and AKI [33]. Endothelial and endothelial surface layer damage can also trigger inflammation and microthrombosis, resulting in kidney injury [34].

Furthermore, both AKI and mortality were associated with transfusion in a dose-dependent manner, with increasing transfusion rates correlating with worse clinical outcomes. These results hint towards the importance of utilizing blood conservation strategies to reduce the need for transfusions of any blood products and challenge the hypothesis that restoration of intravascular circulating volume is feasible with transfusions only [35]. Notably, in our multivariable analysis, PRBC transfusion was the only blood component independently associated with high inflammatory panel alteration, whereas FFP and platelet transfusion were not. This finding is consistent with the concept of transfusion-related immunomodulation (TRIM), whereby stored red blood cells accumulate bioactive mediators and undergo structural changes during storage that promote recipient immune activation and endothelial injury [36].

Certain limitations have to be considered regarding our results. Our observational design cannot establish causality, and the association between transfusion and CLS markers may reflect confounding by indication, as patients with transfusion requirement often have underlying conditions, including hemorrhagic shock, prolonged hypotension, more complex surgery, or longer CPB time that independently promote systemic inflammation as well as endothelial injury. Although we adjusted for established markers of illness severity, overlapping pathophysiological processes and residual confounding may still contribute to the associations observed between transfusion, endothelial biomarkers, fluid shifts, and the derived clustering phenotype. Therefore, our findings should be interpreted as associative and non-causal. Future translational research involving in vitro methods is needed to assess how transfusions affect endothelial cells under inflammatory and non-inflammatory conditions. Additionally, transfusions are hardly administered without a clear indication, raising the question whether the pathology leading to the transfusion or the transfusion itself presenting the culprit. Transfusions administered beyond postoperative day 1 were not captured, so the potential effects of transfusions later in the postoperative course could not be assessed. Although competing risks between inflammation, ICU discharge, and death are possible, their impact in the presented study may be limited as biomarker measurements were obtained on postoperative day 1, before most competing events occurred. Furthermore, the absence of a universally accepted definition or standardized diagnostic criteria for CLS makes the identification of patients with CLS inherently approximate, and is an important limitation of our study. Notably, our definition of CLS is consistent with contemporary literature [11,12,14]. The single-center design of this study likely resulted in more consistent peri- and postoperative management, but could limit external validity of the findings. It is necessary to confirm the generalizability of the findings to other patient populations and healthcare contexts.

In conclusion, our results indicate that transfusions of red blood cells, fresh frozen plasma, or platelets are associated with complications in a dose-dependent manner. We found that transfusion was associated with a pro-inflammatory response among cardiac surgery patients, along with modest but statistically significant fluid shifts consistent with capillary leak. Our study further highlights that transfusion-associated capillary leak was associated with deleterious complications, including acute kidney injury and death. Herein, our data emphasize the importance of carefully considered transfusion in cardiac surgery.

## CRediT authorship contribution statement

All authors made substantial contributions to the conception and design of the study, acquisition of data, or analysis and interpretation of data. JH and JW contributed equally to this work and share last authorship. All authors read and approved the final manuscript.

## Consent for publication

Not applicable.

## Ethics approval and consent to participate

IRB approval was obtained (Freiburg, EK-Nr. 405/18) by the Ethics Committee of the University of Freiburg, Engelbergerstrasse 21, 79106 Freiburg, Germany on January 14, 2019, and complies with the tenets of the Declaration of Helsinki. The study was registered in the German Clinical Trials Registry (DRKS No. 00017057), and written informed consent was obtained from all participants.

## Funding

This work was supported by Förderverein Deutsches Herzzentrum München.

## Availability of data and material

Available from the corresponding author upon reasonable request.

## Declaration of competing interest

No competing interests.
