# Supplementary figures and images for: Transfusion-associated capillary leak in cardiac surgery is linked to adverse postoperative outcomes: a prospective observational study
Source: Ann Intensive Care. 2026 Mar 2;16:100040. doi: 10.1016/j.aicoj.2026.100040 (PMC13045540; doi:10.1016/j.aicoj.2026.100040)

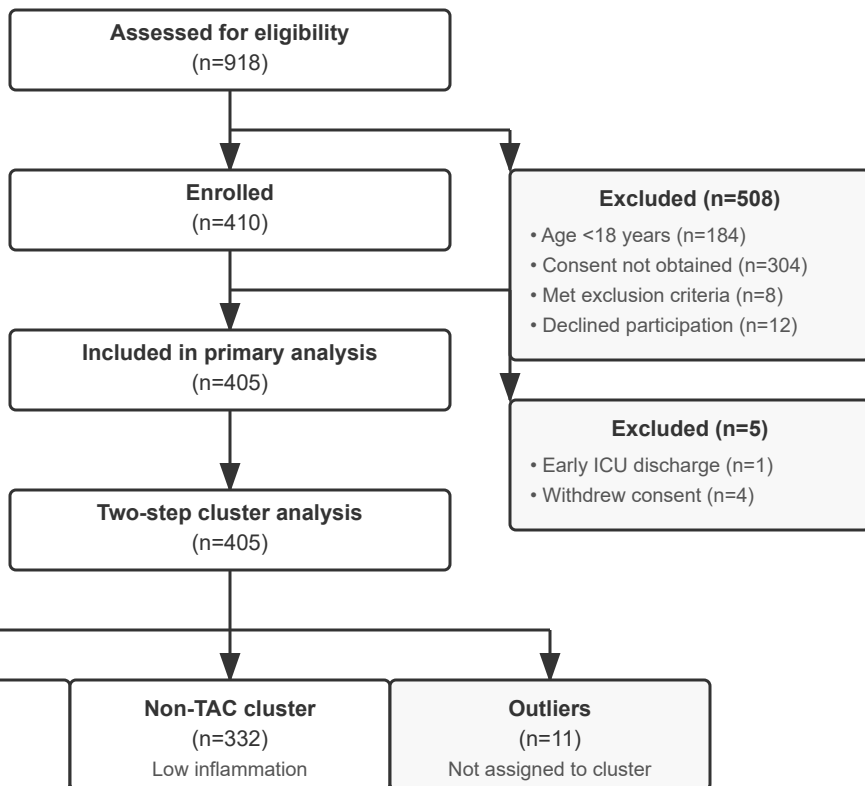

Supplement: Supplementary file 1 [file mmc1.pdf]

**AKI After PRBC Transfusions**

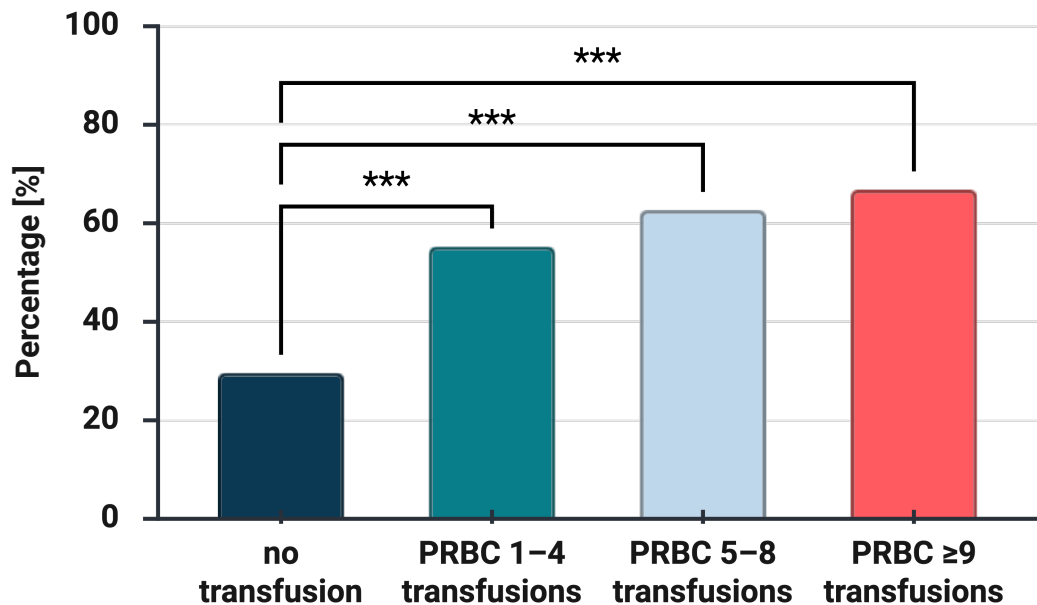

**AKI After FFP Transfusions**

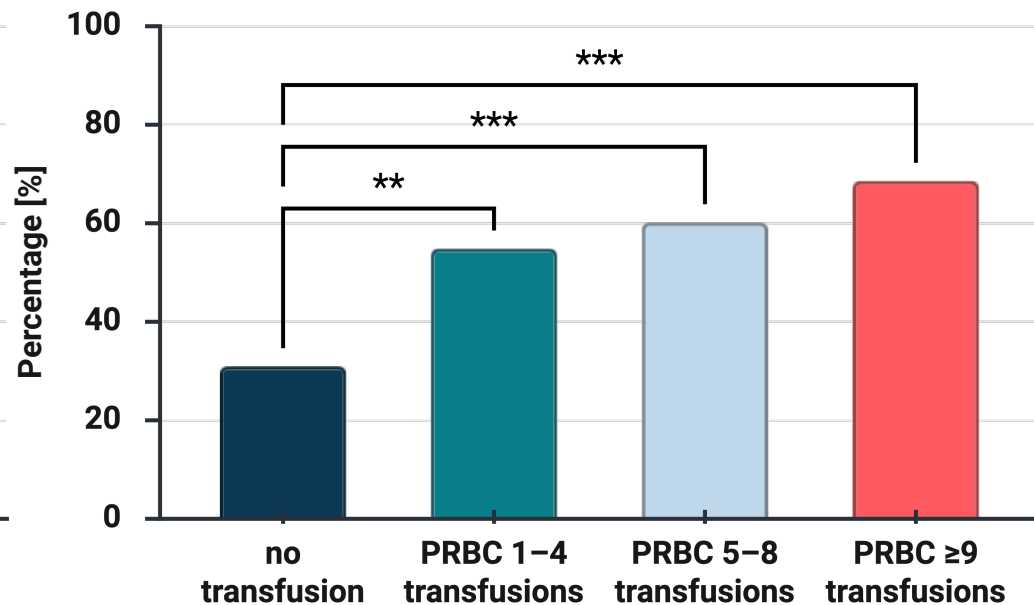

**AKI After Platelet Transfusions**

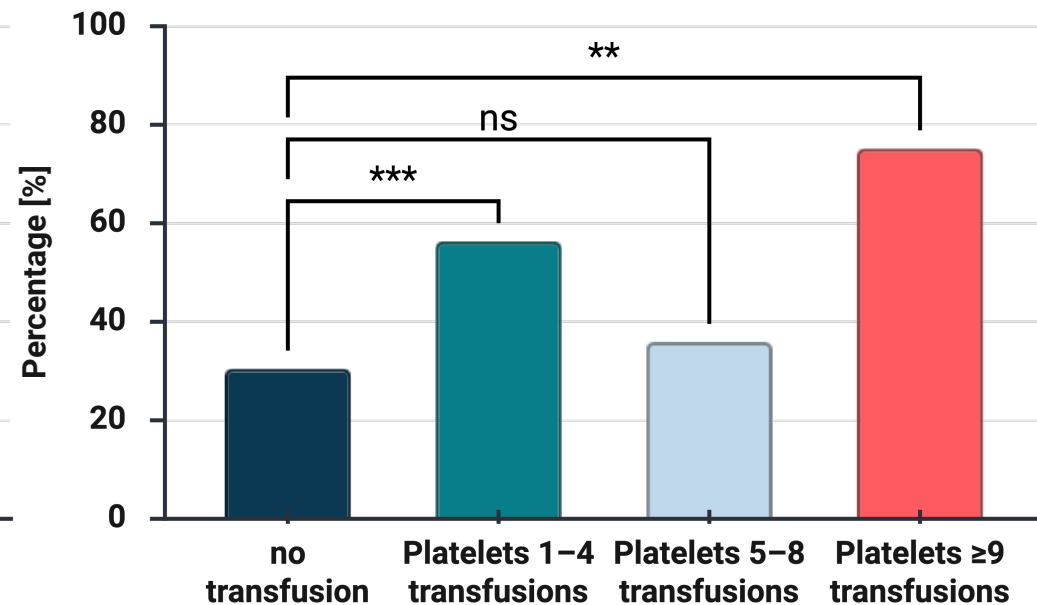

Supplement: Supplementary file 3 [file mmc3.pdf]
