# Supplementary material for: Transfusion-associated capillary leak in cardiac surgery is linked to adverse postoperative outcomes: a prospective observational study
Source: Ann Intensive Care. 2026 Mar 2;16:100040. doi: 10.1016/j.aicoj.2026.100040 (PMC13045540; doi:10.1016/j.aicoj.2026.100040)
Supplement: Supplementary file 2 [file mmc2.docx]

**Supplemental File 2: Transfusion-Associated Capillary Leak in Cardiac Surgery Is Linked to Adverse Postoperative Outcomes**


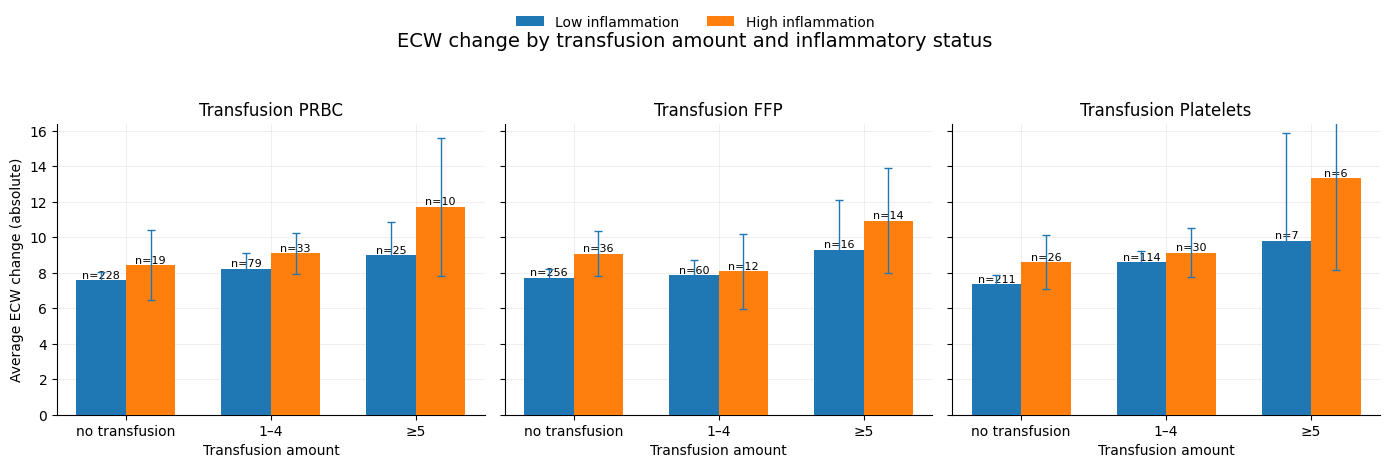


Illustration of the impact of transfusion on ECW changes depending on the inflammatory panel alterations of patients. Average ECW change (absolute) was calculated as ΔECW (POD1–pre-op); higher values indicate an increase in ECW. The overall association between high inflammatory panel alteration and ECW increase was significant (p=0.015, Table 3). Individual pairwise comparisons within transfusion categories were not statistically significant, likely due to limited sample sizes in the high-inflammation subgroups (n=6–33 per cell). Error bars represent 95% confidence intervals.
